# Characteristics of inflammatory and infectious diseases of the pituitary gland in patients undergoing transsphenoidal surgery

**DOI:** 10.1007/s11102-023-01333-4

**Published:** 2023-06-30

**Authors:** Paul Vincent Naser, Penelope Papadopoulou, Jan Teuber, Stefan Kopf, Jessica Jesser, Andreas W. Unterberg, Christopher Beynon

**Affiliations:** 1 Department of Neurosurgery, Heidelberg University, Im Neuenheimer Feld 400, Heidelberg, 69120 Germany; 2 Department of Endocrinology, Heidelberg University, Im Neuenheimer Feld 410, Heidelberg, 69120 Germany; 3 Department of Neuroradiology, Heidelberg University, Im Neuenheimer Feld 400, Heidelberg, 69120 Germany; 4 Department of Neurosurgery, Heidelberg University Hospital, Im Neuenheimer Feld 400, Heidelberg, 69120 Germany

**Keywords:** Transsphenoidal surgery, Pituitary abscess, Pituitary inflammation, Pituitary surgery, Lymphocytic hypophysitis

## Abstract

**Purpose:**

Inflammatory and infectious diseases of the pituitary gland (IIPD) are rare lesions often misdiagnosed preoperatively. Immediate surgery is indicated especially in cases of neurological impairment. However, (chronic) inflammatory processes can mimic other pituitary tumors, such as adenomas, and data on the preoperative diagnostic criteria for IIPD are sparse.

**Methods:**

We retrospectively reviewed medical records of 1317 patients who underwent transsphenoidal surgery at our institution between March 2003 and January 2023. A total of 26 cases of histologically confirmed IIPD were identified. Patient records, laboratory parameters, and postoperative course were analyzed and compared with an age, sex, and tumor volume-matched control group of nonfunctioning pituitary adenomas.

**Results:**

Pathology confirmed septic infection in ten cases, most commonly caused by bacteria (3/10) and fungi (2/10). In the aseptic group, lymphocytic hypophysitis (8/26) and granulomatous inflammation (3/26) were most frequently observed. Patients with IIPD commonly presented with endocrine and/or neurological dysfunction. No surgical mortality occurred. Preoperative radiographic findings (cystic/solid tumor mass, contrast enhancement) did not significantly differ between IIPD and adenomas. At follow-up, 13 patients required permanent hormone substitution.

**Conclusion:**

In conclusion, correct preoperative diagnosis of IIPD remains challenging, as neither radiographic findings nor preoperative laboratory workup unequivocally identify these lesions. Surgical treatment facilitates decompression of supra- and parasellar structures. Furthermore, this low-morbidity procedure enables the identification of pathogens or inflammatory diseases requiring targeted medical treatment, which is crucial for these patients. Establishing a correct diagnosis through surgery and histopathological confirmation thus remains of utmost importance.

## Introduction

Transsphenoidal surgery (TSS) is the most common neurosurgical approach for tumors of the sellar region, as it offers direct access to the pituitary region and is associated with a low perioperative risk [1]. Approximately 70–80% of surgeries are performed for pituitary adenomas [2], a group subdivided into hormone-secreting, functional, and inactive non-functioning subtypes. Other benign lesions of the sellar region include Rathke’s cleft cysts, craniopharyngiomas, and meningiomas, which together constitute approximately 15% of cases. The remaining pathologies – thus far predominantly presented as case reports - are malignant sellar tumors [3], metastasis [4], and infectious and inflammatory pituitary lesions (IIPD) [5].

IIPD subsumes a heterogenous group of septic infections resulting in abscess and aseptic pituitary lesions. Primary pituitary abscesses occur in an otherwise healthy pituitary gland and account for about 70% of cases [6]. The causal root of primary pituitary abscesses remains clouded, but an association with immunosuppression, diabetes mellitus, or pregnancy has been described in the literature [7]. Secondary pituitary abscess accounts for about 30% of the cases and typically occurs in patients with pre-existing pituitary lesions, most commonly adenomas [6], or iatrogenic, e.g. following pituitary surgery [8]. They can arise either from continuous spread from nearby structures, or hematogenous dissemination from distant sites. Sepsis, meningitis or sinusitis are frequently observed in secondary pituitary abscess [6, 9].

Contrary to pituitary abscesses, aseptic inflammation of the pituitary gland subsumes several autoimmune disorders, e.g. lymphocytic, granulomatous, IgG_4_-associated, xanthomatous, and necrotizing pituitary inflammations [10, 11]. The lymphocytic subtype is the most common (70%) aseptic pituitary inflammation. The exact etiology is unknown, but it most frequently occurs in the last trimester of pregnancy and the peripartal period. Lymphocytic hypophysitis is associated with a benign prognosis, and patients usually respond well to corticoid and immunosuppressive therapy [12]. Granulomatous hypophysitis may occur primarily, restricted to the pituitary gland or secondary to systemic diseases such as sarcoidosis, tuberculosis, or Morbus Wegener. Patients typically present with more severe clinical symptoms, and the prognosis, even with optimal immunomodulatory treatment, is less favorable than for the lymphocytic subtype [13, 14].

The preoperative diagnosis of inflammatory pituitary lesions remains challenging, as data on these rare pathologies is largely derived from case reports or small clinical series.

## Methods

We retrospectively reviewed the patient records of our department at Heidelberg University Hospital between 03/2003 and 01/2023 and identified patients with the specific surgical code for TSS. Final pathology results were reviewed for all cases, and patients diagnosed with inflammation, hypophysitis, infection, or abscess were included (IIPD). An age-, gender- and tumor-size-matched control group of patients with nonfunctioning pituitary adenoma (NFPA) was randomly selected. For both groups, biographic data, as well as data on the preoperative presentation (neurological/endocrine status), history of infection or immune suppression, as well as pre- and postoperative laboratory parameters (Na, K, Ca, Cl, Cr, GFR, UA, Gluc, CRP, PCT, Copeptin, leukocyte count, erythrocyte count, hemoglobin, hematocrite, MCV, MCH, MCHC, RDW, thrombocyte count, % hypochromic erythrocytes, Quick, INR, aPTT, fibrin degradation product, Factor XIII, Fibrinogen, TSH, fT3, fT4, PrL, FSH, LH, Progesterone, Estradiol, Testosterone, ACTH, STH, IGF-1, Cortisol) were analyzed. Preoperative magnetic resonance imaging was analyzed using Origin Server 3.2 (Brainlab GmbH, Munich, Germany). Statistical analysis and figure design were performed with Prism 9 (Graphpad Software, Boston, MA, USA) and Adobe Illustrator (Adobe Inc., San Jose, CA, USA).

## Results

**Fig. 1:**
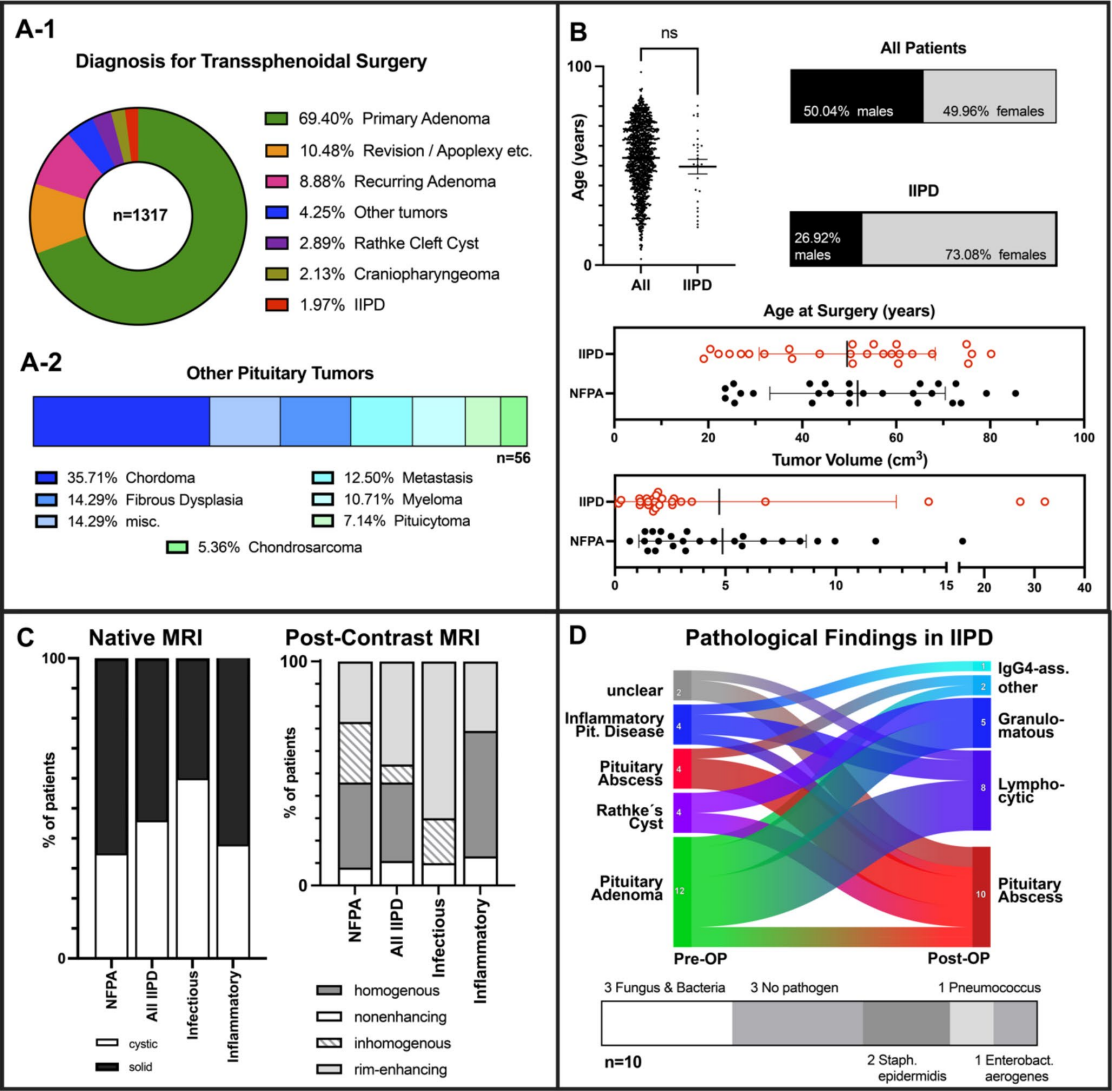
**A**: Overview of transsphenoidal surgeries at our center (A-1), and frequencies of rare and malignant pituitary tumor entities (A-2). **B**: Age and Sex distributions of the entire patient collective (top) and subgroups NFPA and IIPD (bottom). **C**: Radiographic findings in IIPD **D**: Top: Sankey chart denoting pre- (left) and postoperative (right) diagnosis in IIPD. Types of pathogens identified in infectious pituitary disease (bottom)

**Fig. 2:**
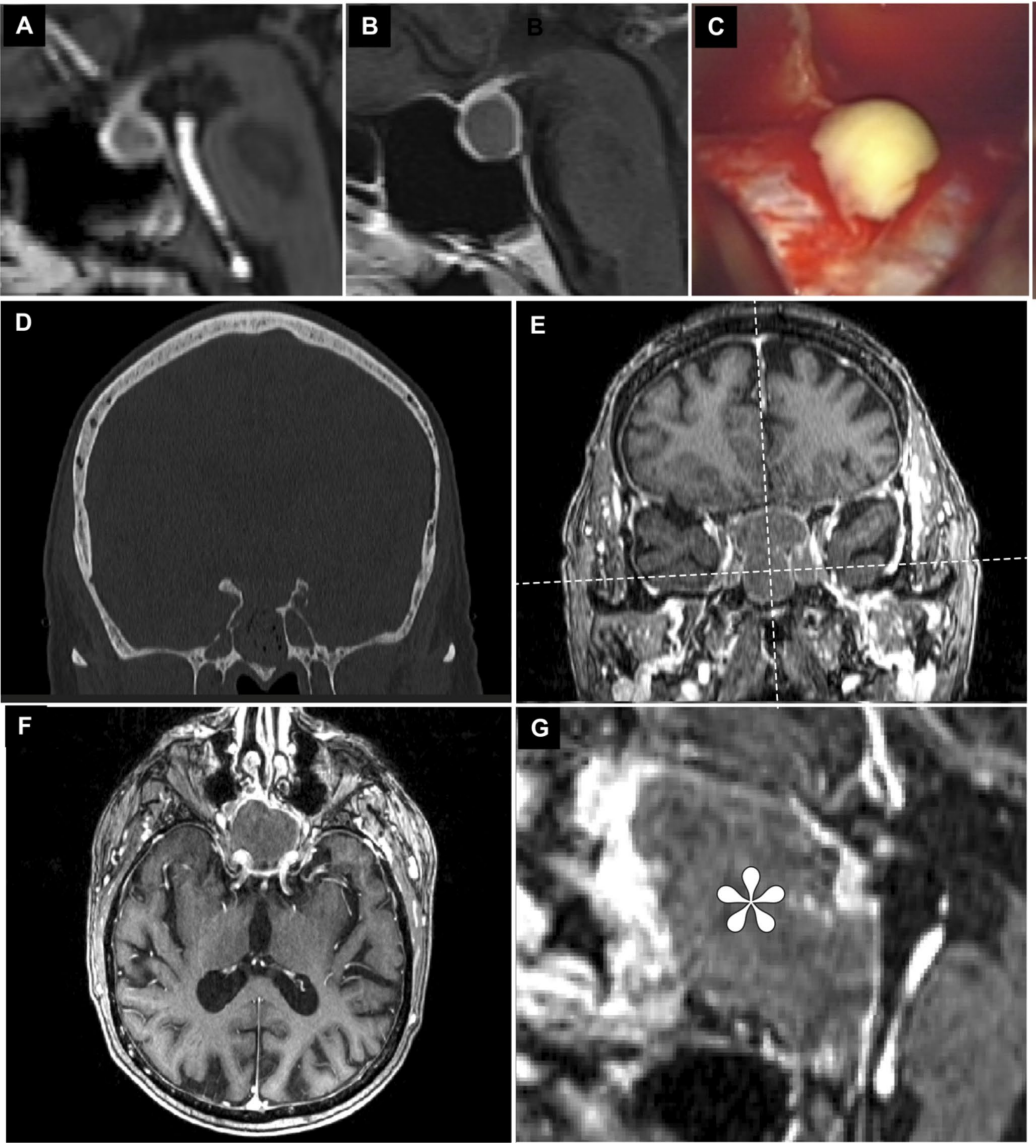
Illustrative Case 1: Post-contrast MRI four months prior to admission (**A**) and at admission (**F**), note the signs of central pontine myelinolysis. **C**: Intraoperative putrid secretion encountered during surgery. Illustrative Case 2: Partial osseous destruction revealed in the bone-window cranial CT (**D**). CE-MRI at admission revealed the tumor extending through the sphenoid sinus encompassing the right ACI (**E**-**F**). Enlarged view of the sagittal plane, abscess denoted with asterisk. Planes for (**F**) and (**G**) are denoted in (**E**)

26 patients (73% female, 27% male, mean age 49.6y) with histologically confirmed IIPD were identified from our database of surgeries performed from March 2003 until January 2023 (50% female / 50% male; mean age 54 years). More than three-quarters of patients underwent surgery for pituitary adenoma, both primary and recurring (Fig. <link rid="fig1”>1</link>A-1). Other indications for surgery were craniopharyngioma, revision surgery, e.g. for cerebrospinal fluid fistula, or malignant tumors (Figs. 1A and 2). IIPD represents the smallest subgroup of our collective (Fig. <link rid="fig1”>1</link>A-1). Regression analysis revealed a significant increase in both the absolute number of TSS conducted (F = 33.9, p < 0.001) as well as in the fraction of IIPD encountered (F = 10.78; p = 0.004) between 2003 and 2023. Of those patients, ten were diagnosed with florid infection, either with or without a confirmed pathogen (Fig. 1D). Within the aseptic group, eight patients were diagnosed with lymphocytic hypophysitis (75% female, mean age 57.4 years). Five cases of granulomatous hypophysitis (60% female, mean age 52.2 years) and one 60- year old male patient with IgG_4_-associated hypophysitis were identified. In two cases, clinical findings, as well as pathological results, showed pituitary inflammation, however inconclusive as to the exact subtype. Preoperatively, pituitary abscess and inflammatory pituitary disease were correctly suspected in four cases, respectively; other putative preoperative diagnoses included Rathke’s cleft cyst and pituitary adenoma (Fig. 1D).

All patients were originally scheduled for transsphenoidal surgery (TSS). In one case of inflammatory pituitary disease, the decision was made immediately preoperatively to approach the lesion via frontotemporal craniotomy for anatomical reasons. This case was excluded from the further analysis of surgical technique. Surgical time was 133 ± 58 min in NFPA patients and 113 ± 79 min in IIPD (p = 0.31). Intraoperative MRI was conducted in 77% of NFPA cases and 38% of IIPD cases. Following MRI, in six NFPA and no IIPD cases, additional tumor was removed. Intraoperative frozen section diagnostic was conducted in 19% of NFPA and 23% of IIPD cases.

**Table 1:** *Top*: Patient history and symptoms recorded at presentation. Columns denote the prevalence in NFPA, IIPD, and the subgroups of inflammatory and infectious pituitary disease. Shaded columns indicate p-values (X^2^-test). *Bottom*: Preoperative laboratory parameters for NFPA, IIPD, and the subgroups of inflammatory and infectious pituitary disease. Shaded columns indicate p-values (t-test). P-values below 0.05 are marked in bold font

		NFPA	IIPD	NFPA vs IIPD	Infectious pituitary disease	NFPA vs Infectious pituitary disease	Inflammatory pituitary disease	NFPA vs Inflammatory pituitary disease
Patient History	Immune Suppression	11.6%	23.1%	0.272	20.0%	0.512	25.0%	0.256
	Infection	7.69%	26.9%	0.067	30.0%	0.083	25.0%	0.120
	Pregnancy	0.0%	3.85%	0.313	0.0%	-	6.25%	0.197
Symptoms at presentation	Endocrine dysfunction	19.2%	57.7%	**0.004**	20.0%	0.958	81.3%	**< 0.0001**
	Neurological Dysfunction	69.2%	61.5%	0.560	80.0%	0.518	50.0%	0.213
	Headache	42.3%	38.5%	0.778	60.0%	0.341	25.0%	0.256
	Misc. neurol. Symptoms	23.1%	23.1%	-	30.0%	0.667	18.8%	0.740
	Oculomotor paresis	15.4%	26.9%	0.308	50.0%	**0.032**	12.5%	0.795
	Vision loss	48.0%	19.2%	**0.029**	10.0%	**0.036**	25.0%	0.141
Preoperative laboratory parameters	Sodium (135–146 mmol/l)	140.7	138.3	**0.035**	137.4	**0.045**	138.8	0.116
	Potassium (3.4-5 mmol/l)	3.737	4.034	**0.003**	3.966	**0.044**	4.076	**0.007**
	C-reactive Protein (< 5mg/l)	11.86	20.49	0.420	44.44	**0.0499**	5.519	0.094
	Procalcitonin (< 0.05 ng/ml)	0.160	1.530	-	3.010	-	0.050	-
	Leukocyte count (4–10 /nl)	11.85	8.903	**0.003**	8.917	**0.037**	8.894	**0.007**
	Copeptin (< 10 pmol/l)	7.250	5.205	0.448	11.24	0.067	3.864	0.055
	Thyroid stimulating hormone (0.4-4 mU/l)	1.265	1.280	0.975	1.091	0.690	1.415	0.794
	Prolactin (43–375 mU/l)	234.8	633.7	0.084	665.2	0.130	598.6	**0.049**
	Cortisol (56–200 ng/ml)	502.7	168.6	**0.004**	162.6	**0.033**	172.0	**0.019**

Age and sex distribution were markedly different for IIPD (Fig. 1B) compared to all patients who underwent TSS. We thus extracted an age-, sex- and a tumor-volume-matched control group of nonfunctioning pituitary adenomas (NFPA) to compare IIPD against. IIPD patients, especially patients with inflammatory pituitary disease, showed a higher rate of immune suppression than NFPA patients (Table 1). Correspondingly, prior infection was elevated in IIPD, in particular in those cases of florid abscess. Of the entire collective, only one patient was treated surgically during the peripartal period (3 days postpartum), she was diagnosed with lymphocytic hypophysitis.

At admission, IIPD patients primarily presented with neurological deficits and/or endocrine dysfunction (Table 1). The rate of endocrine dysfunction was significantly higher in IIPD than NFPA patients, an effect primarily attributable to the subgroup of inflammatory pituitary lesions. Vision loss, the most common neurological symptom in adenoma patients, was only encountered in a minority of IIPD patients (Table 1).

Preoperative tumor volumes were similar in IIPD and NFPA (4.872 cm^3^ vs. 4.722 cm^3^; p = 0.93). Preoperative radiographic findings (cystic/solid tumor mass, contrast enhancement) did not significantly differ between IIPD, their subgroups when compared to NFPA (Fig. 1C). Linear regression analysis revealed tumor volume to be significantly associated with vision loss in NFPA (F = 11.14; p = 0.003), but not IIPD patients.

Of the forty preoperative laboratory parameters analyzed in this study, especially electrolytes and cortisol were significantly altered in IIPD. C-reactive protein and procalcitonin but not the leukocyte count were elevated in cases of pituitary abscess (Table 1).

One IIPD patient developed new neurological symptoms (mild deterioration of vision) postoperatively, which had completely remised at follow-up. No deaths were recorded. The mean postoperative stay was 7.2 ± 3.7 days for adenoma patients. IIPD patients, on average, remained hospitalized one additional day (8.2 ± 3.3 days), especially those with pituitary infection (8.8 ± 4 days), the differences, however, were not statistically significant.

The mean follow-up was 713 days for IIPD and 1004 days for NFPA (p = 0.49). At follow-up, the rate of hormone replacement in NFPA patients was low (6/26), with one patient already requiring substantial supplementation at presentation. 12 IIPD patients were on hormone replacement therapy at follow-up, especially inflammatory pituitary disease patients (56%; p = 0.029). Lesion size or preoperative hormonal imbalance was not significantly associated with hormone replacement at follow-up. 11 IIPD, and 11 NFPA patients had experienced remission of preoperative neurological symptoms. None of the IIPD patients experienced recurrence or required additional surgery. In one NFPA patient, tumor recurrence was observed during routine radiographical controls and surgically treated two years after the initial surgery.

### Illustrative case 1

A twenty-four-year-old female patient was referred to our center after treatment of severe hyponatremia at an outside institution. Due to an Addisonian crisis four months before referral, the patient had developed central pontine myelinolysis. Initially, a pituitary mass was diagnosed on the cranial MRI (Fig. 2A). At presentation, the patient had recovered well from the pontine myelinolysis but reported mild subjective visual impairment and recurring headaches. Following discharge from the primary hospital, she reported recurring sub febrile episodes that were treated intermittently with antibiotics by her general practitioner, the cause of which remained unresolved. She was on thyroid and corticoid substitution. Neurological examination revealed mild ataxia, the visual deficit could not be objectified. High-resolution contrast-enhanced MR-imaging was conducted at our center (Fig. 2B). Laboratory parameters, especially infectious markers, were normal. TSS was recommended, as the lesion had increased in size compared with the prior imaging. Intraoperatively, a florid putrid pituitary abscess was encountered (Fig. 1C), which was completely drained. The patient tolerated the surgery well and was discharged home on the sixth postoperative day. Microbiology revealed infection with *Staphylococcus epidermidis*, which was treated with cefuroxime for six weeks postoperatively. At follow-up (2 years), the patient was in excellent health with complete remission of the subjective visual impairment, as well as the headaches and ataxia.

### Illustrative case 2

A 75- year old male presented with recurring diplopia. Additionally, his general condition had significantly deteriorated over the past years. He was on metformin and insulin for type 2 diabetes, ruxolitinib, and prednisolone for polycythemia vera. He was anticoagulated with rivaroxaban for atrial fibrillation and congestive heart failure was treated with ramipril and torasemide. He reported oculomotor dysfunction, which had affected the left eye the previous year, spontaneously remitting after several weeks, recurred eight weeks prior, now affecting the right eye. The patient reported complete anosmia for several years and recently recurring episodes of vertigo. Neurologically, the patient exhibited complete anosmia, as well as right-sided ophthalmoparesis with diplopia. No additional deficits were noted. Preoperative laboratory testing was unremarkable, showing mild hypercortisolism under prednisolone and especially no increased infectious markers.

Cranial computed tomography (Fig. 2F) suggested an extensive lesion affecting the ethmoidal cells, the sphenoid sinus, and reaching the sellar structures. Gadolinium-enhanced cMRI revealed an extensive lesion encompassing most of the sphenoid and the ethmoidal cells. Neuroradiologically, putative diagnoses were mucocele or atypically hypovascular meningioma. Transsphenoidal surgery was scheduled for decompression of the skull base and bioptic confirmation of the entity. Intraoperatively, massive putrid, necrotic tissue engulfing the mostly lytic osseous surrounding was encountered. Fresh frozen pathology revealed fungoid hyphae. Intraoperative MRI confirmed total resection and sufficient decompression. Antibiotic therapy was immediately started with ceftriaxone and liposomal amphotericin B, and adapted on POD 4 to isavuconazole + ceftazidime after microbiology confirmed *Aspergillus fumigatus* and two separate entities of *Pseudomonas aeruginosa*. No new neurological deficits were observed postoperatively. The patient was transferred for further geriatric therapy on POD 8. The anti-infective therapy was deescalated after three weeks to ciprofloxacin + isavuconazole and continued for six months. Nine months postoperatively, the patient reported complete remission of the ophthalmoparesis. Endocrinological testing confirmed sufficient cortico-, thyreo-, galacto-, and gonadotropic function, and all supplementation could be discontinued.

## Discussion

### Transsphenoidal surgery

Transsphenoidal surgery (TSS), as first described over 100 years ago, has been the standard approach to sellar lesions in general and pituitary lesions especially [15]. Recently, the field has seen a shift from microsurgical to endoscopic TSS [16]. Both techniques are considered equally successful and are associated with low perioperative risk when performed in experienced centers [1, 17–19]. We almost exclusively operate microsurgically, and we have found the routine use of intraoperative MRI exceedingly valuable, resulting in high rates of complete tumor removal at the primary surgery [20]. In cases of unclear preoperative diagnosis or when unexpected tissue is observed intraoperatively, we utilize frozen pathology. Surgical durations were slightly lower in IIPD patients, likely the result of rapid lesion drainage in case of liquid pus in cases of putrid infection or the surgeon’s restriction to obtaining a biopsy in cases of suspected inflammatory pituitary disease. Additionally, when putrid infection was encountered, intraoperative MRI was forgone, further reducing operative time.

### Aseptic hypophysitis

Diagnosing inflammatory pituitary disease is exceedingly difficult, with patients frequently presenting with a range of endocrinological symptoms. TSS has been surpassed as first-line therapy by steroid treatment when pituitary inflammation is suspected. This can lead to complete remission, considered as confirmation of the diagnosis. Due to the disease’s inconclusive preoperative presentation, exact diagnosis can, however, only be obtained via biopsy [21].

Within our subgroup of non-infectious inflammatory pituitary patients, lymphocytic hypophysitis was observed most frequently. From the literature, a frequent association with the peripartal period was expected [12]. In our dataset, however, only one patient was diagnosed peri partum (three days after delivery). This may call into question the heavy reliance on “pregnancy” as a diagnostic criterium [22]. Preoperative diagnosis is further challenged by inconclusive preoperative imaging. Radiographically, inflammatory pituitary lesions often mimic pituitary adenomas, frequently showing isointense with slight heterogeneity on native T1 and homogenous gadolinium enhancement [23]. Parasellar T2 hypointensity was suggested to indicate lymphocytic hypophysitis [24]. Additionally, novel scores have been proposed for the diagnosis of lymphocytic hypophysitis [22]. Once reliably validated, applying these scores and primary medical treatment may lead to a marked reduction in the number of surgically treated patients.

Granulomatous hypophysitis is another rare subtype of inflammatory lesions we encountered in less than 0.5% of surgeries. Radiographically, those tumors closely mimic pituitary macroadenomas, and due to their rare occurrence, our knowledge about this pathology is limited [25, 26]. Cases of the granulomatous subtype responding to systemic corticosteroids without surgery have been described [13, 14]. It should be noted that in these cases, the diagnosis merely rests on clinicoradiographic findings without pathology. In our series, we did not encounter granulomatous hypophysitis secondary to systemic granulomatosis, e.g. Morbus Wegener or tuberculosis, so all described cases are considered primary granulomatous hypophysitis.

Patients diagnosed with inflammatory pituitary disease had higher rates of preoperative immune suppression, a finding that may support the theory of aseptic hypophysitis resulting from an altered immunological state [26].

Diagnosing inflammatory pituitary disease preoperatively remains extremely difficult due to these disorders’ clinical and radiological ambiguity. While in neurologically asymptomatic cases, corticosteroid treatment may be effective, once the inflamed pituitary excises a mass effect causing neurological symptoms, surgical excision should be performed, enabling besides decompression the pathological diagnosis and targeted treatment.

### Infectious pituitary disease / pituitary abscess

Although first reported over 150 years ago, pituitary abscess remains an elusive pathology. Most cases are reported in small retrospective series and single case reports [7, 27, 28]. Similar to our series (0,76%), the incidence has been approximated between 0.7 and 1% of all TSS. In total, approximately 500 cases of pituitary abscess have been reported in the literature [7, 27, 28].

Patients with pituitary abscess typically presented with neurological deficits, such as strong headaches or oculomotor palsy. Contrary to NFPA patients, only one of the pituitary abscess patients reported visual deficits, whereas the rate of oculomotor palsy was significantly higher in this group. This may be due to the tissue reaction caused by the infectious process in the sella affecting the cranial nerves within the cavernous sinus before the mass effect of the lesion affects the optic tract. The illustrated cases further underscore the breadth of possible clinicoradiological presentations.

Preoperative laboratory parameters such as leukocyte count or CRP have been reported as inconclusive in the preoperative diagnosis of a pituitary abscess. We found CRP to be slightly elevated in our collective of infectious pituitary lesions. The novel bacterial marker PCT [29] was markedly elevated in patients diagnosed with abscess. Unfortunately, due to its non-standard assessment in our preoperative laboratory panel, this parameter was unavailable for all but one NFPA patient, prohibiting statistical analysis.

Colleagues from China recently published a series of 12 patients, who developed pituitary abscess following TSS at peripheral hospitals [30]. None of our patients had undergone prior cranial/transnasal surgery, and a history of infection was likewise not significantly associated with either inflammatory or infectious pituitary disease in our collective.

Similar to some cystic adenomas and Rathke’s cleft cysts, pituitary abscess presented predominantly as a cystic, rim-enhancing lesion in MR imaging, a finding that seems characteristic of this pathology [31]. While in rare cases, antibiotic treatment alone has been utilized curatively, surgical abscess drainage remains undoubtedly the standard of care for these patients. We routinely administered adjuvant antibiotic treatment for a minimum of six weeks postoperatively and could not observe any instances of abscess recurrence.

## Limitations

We performed a retrospective analysis on patients who underwent transsphenoidal/transnasal surgery. Due to our patient inclusion based on the surgical code, patients operated via craniotomy may not have been included. Further, a complete preoperative laboratory workup, including pituitary hormones and sensitive infection markers (e.g. PCT) was unavailable for some patients. Additionally, detailed medication at follow-up was inconsistently reported, potentially misrepresenting the rate of postoperative hormone replacement.

## Conclusion

Infectious and inflammatory pituitary pathologies are exceedingly rare lesions of the pituitary gland that are often mistaken for adenomas due to their similar clinical and radiological characteristics. Traditional infection markers are not able to reliably predict pituitary abscess, while newer, sensitive markers may be indicative of florid abscesses. New methodologies have been proposed but not yet clinically validated to distinguish aseptic hypophysitis preoperatively. More studies on larger cohorts are warranted to improve treatment modalities in respective patients.

## Data Availability

Raw data are available from the corresponding author upon reasonable request.
